# The Efficacy of Pramipexole, a Dopamine Receptor Agonist, as an Adjunctive Treatment in Treatment-Resistant Depression: An Open-Label Trial

**DOI:** 10.1100/2012/372474

**Published:** 2012-08-01

**Authors:** Hiroaki Hori, Hiroshi Kunugi

**Affiliations:** Department of Mental Disorder Research, National Institute of Neuroscience, National Center of Neurology and Psychiatry, 4-1-1 Ogawahigashi, Kodaira, Tokyo 187-8502, Japan

## Abstract

Dopaminergic dysfunction is implicated in the pathophysiology of treatment-resistant depression. Although the efficacy of adjunctive pramipexole treatment has been demonstrated in treatment-resistant bipolar depression, such data are scarce for major depressive disorder (MDD). We recruited 17 patients with DSM-IV major depressive episode who have failed to respond to previous treatment with a selective serotonin reuptake inhibitor. Five patients were diagnosed as having bipolar II disorder and 12 as having unipolar MDD. Patients were monitored at an ambulatory care facility every two weeks until 12 weeks. Pramipexole was added to existing medication. Depression severity was assessed with the Hamilton Depression Rating Scale 21-item version (HDRS-21). The mean maximum dosage of pramipexole was 1.6 mg (SD 0.9). The HDRS-21 total score decreased from 19.4 (SD 3.8) at baseline to 7.2 (SD 5.4) at endpoint (*P* < 0.000001). Twelve patients (71%) were responders based on the definition of 50% or more reduction in the HDRS-21 score. Ten patients (59%) remitted (HDRS-21 total score at endpoint <8). These results were almost unchanged when the sample was confined to patients with MDD. No serious adverse events were observed. Our findings indicate that pramipexole augmentation therapy may be effective and well tolerated in refractory depressed patients.

## 1. Introduction

It is well known that a significant proportion of patients with major depressive disorder fail to achieve remission with standard antidepressant therapies, even when optimally delivered. Such a condition is called treatment-resistant (or refractory) depression and represents a major challenge in everyday practice. Treatment-resistant depression can be classified into different stages based on the degree of treatment resistance; Thase and Rush [1] defined stage I treatment-resistant depression as the persistence of significant depressive symptoms, despite at least one adequate trial with one major class of antidepressant, stage II as stage I resistance plus failure of an adequate trial with an antidepressant in a different class from that used in stage I, and stage III as stage II resistance plus failure of an adequate trial with a tricyclic antidepressant. 

As dopamine is involved in the regulation of motivation, volition, interest/pleasure, and attention/concentration, all of which are likely to be impaired in depressed patients, reduced dopamine neurotransmission is implicated in the pathophysiology of depression and thought to play an important role in the treatment-resistant depression [2]. Supporting this, preclinical studies have demonstrated the effectiveness of dopamine agonists in depression [3, 4], and we also reported antidepressant-like and anxiolytic-like effects of cabergoline in rats [5]. It would therefore be reasonable to assume that depressed patients who have not responded to multiple serotonergic and noradrenergic antidepressants may benefit from dopaminergic agents. 

There are six dopamine agonists currently used in clinical practice mainly for Parkinson's disease: bromocriptine, cabergoline, pergolide, talipexole, ropinirole, and pramipexole. Ergot alkaloids (bromocriptine, cabergoline, and pergolide) can cause serious, albeit rare, adverse events including valvular heart diseases whereas nonergot dopamine agonists (talipexole, ropinirole, and pramipexole) do not have such an effect on cardiac valves. Among the latter, pramipexole, a D_2_/D_3_ receptor agonist approved for the treatment of Parkinson's disease and restless legs syndrome, has been demonstrated to have antidepressant efficacy as an adjunctive treatment in treatment-resistant bipolar depression in two randomized placebo-controlled trials [6, 7]. 

On the other hand, evidence for efficacy of dopamine agonists in the treatment of refractory unipolar major depressive disorder (MDD) is scarce. To our knowledge, six studies have investigated the possible effect of adjunctive dopamine agonists in the treatment of refractory depression [8–13]. These studies have generally found marked improvement in depressive symptoms [8–11, 13]; however, most of these studies targeted stage I treatment-resistant depression, with only one study for stage II refractory depression [13]. The latter with an open-label design examined efficacy of adjunctive pramipexole in the treatment of 10 patients with stage II refractory depression during an 8-week follow-up period and showed substantial effect of pramipexole [13]. It is thus suggested that pramipexole augmentation, among various dopamine agonists, may be a worthwhile option for refractory depression. However, more studies are needed to clarify the efficacy of adjunctive pramipexole in the treatment of refractory depression. 

In the present open-label trial, we aimed to examine the efficacy and safety of pramipexole as an adjunctive treatment in patients with treatment-resistant depression. 

## 2. Methods

### 2.1. Study Design

From August 2009 to February 2011, we conducted a 12-week open trial of pramipexole augmentation in treatment-resistant depression at the National Center of Neurology and Psychiatry (NCNP) Hospital, Japan. Seventeen patients diagnosed as having DSM-IV major depressive episode were recruited from the outpatient clinic of the NCNP Hospital or from community through advertisements in free local magazines and our website announcement. All of the community patients had been regularly attending to their nearby hospital or clinic before the participation in the present trial. Diagnosis was made based on the DSM-IV criteria [14] by an experienced psychiatrist. 

Eligible subjects were those who had persistence of significant depressive symptoms as defined by the total score on the Hamilton Depression Rating Scale 21-item version (HDRS-21) [15] of equal to or greater than 15, despite previous 6 weeks or more treatment with adequate dose of at least one selective serotonin reuptake inhibitor. All but one of the patients turned out to have failed to respond to multiple antidepressant trials, that is, stage II or III treatment-resistant depression according to the classification of Thase and Rush [16]. Exclusion criteria were being under 18 or over 64 years old, being pregnant, having a prior medical history of central nervous system disease or severe head injury, having a physical illness that could interfere with the present study, and exhibiting marked suicidality. Those who needed to drive a car were also not eligible because pramipexole is associated with a potential risk of sleep attack. 

The protocol was approved by the Institutional Review Board of the NCNP, and the present trial was conducted in accordance with the Declaration of Helsinki and Good Clinical Practice Guidelines. After the nature of the study procedures had been fully explained, written informed consent was obtained from every subject. 

### 2.2. Treatment

Patients visited the ambulatory care facility of the NCNP Hospital every two weeks up until 12 weeks. Pramipexole was added to each patient's current medication, with the initial dosage of 0.25 mg/day. Dosages were then titrated on case-by-case basis (up to 3 mg daily when needed). Other medication was essentially kept unchanged during the 12-week trial period except for a minor change of sleeping medication. 

### 2.3. Assessments

Adherence to medication was ascertained by clinical interview. Depressive symptoms were assessed with the HDRS-21. Clinical status was assessed with the Clinical Global Impression of Severity (CGI-S) and Improvement (CGI-I) scales [17]. These assessments were made at each visit (i.e., every two weeks). Response to treatment was defined as a 50% or more reduction in the HDRS-21 total score from baseline to endpoint. Remission was defined as a score of 7 or less on the HDRS-21 at endpoint. Safety was determined by adverse event monitoring through clinical observation/interview (at each visit) as well as objective examinations including blood test, urinalysis, and electrocardiogram (at the first, 4-week, and 12-week visits).

### 2.4. Statistical Analysis

Averages are reported as means ± SD (standard deviation). All analyses were performed on the intent-to-treat basis, with the conservative last observation carried forward (LOCF), in patients with at least one available follow-up assessment. The paired *t*-test and the Wilcoxon signed-rank test were used to compare baseline and LOCF results of the HDRS and CGI-S, respectively. Statistical significance was set at two-tailed *P* < 0.05. Analyses were performed using the Statistical Package for the Social Sciences (SPSS) version 18.0 (SPSS Japan, Tokyo). 

## 3. Results

### 3.1. Baseline Demographics and Clinical Characteristics 

Baseline demographics and clinical variables of the sample are summarized in Table 1. Seven males and 10 females, with mean age of 36.2 were enrolled. Of the total 17 patients with a current major depressive episode, 12 had MDD and 5 had bipolar II disorder (Table 2). Of the 12 MDD patients, 3 had comorbid dysthymic disorder. According to the guideline of Thase and Rush [16], one patient was classified as stage I treatment-resistant depression, 3 as stage II, and 13 as stage III. 

The mean dosage of pramipexole at endpoint was 1.6 ± 0.9 mg/day. The maximum dosage was also 1.6 ± 0.9 mg/day. Seven patients were given relatively high dosages (i.e., equal to or greater than 2.0 mg/day), of whom four required the maximum dosage that we set at 3 mg/day. 

### 3.2. Efficacy 

**Figure 1:**
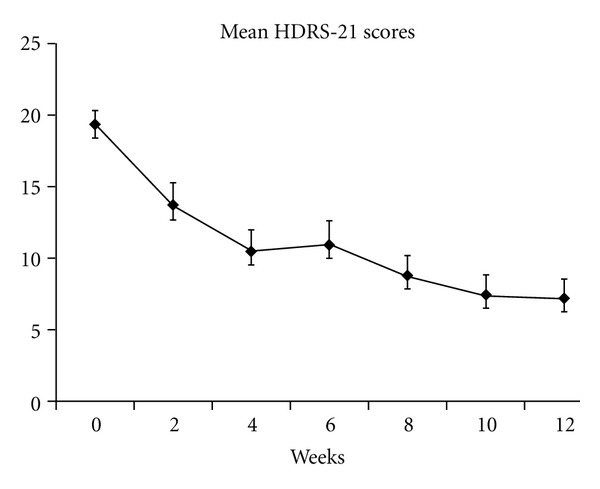
Mean scores over time on the Hamilton Depression Rating Scale (HDRS) 21-item version of the sample (*n* = 17), based on the intent-to-treat analysis. Error bars represent standard errors of the mean.

As shown in Table 2, 12 patients (70.6%) were considered to be responders based on the definition of 50% or more reduction in the HDRS-21 score from baseline to endpoint. Ten patients (58.8%) remitted based on the definition of HDRS-21 score equal to or less than 7. The HDRS-21 total score decreased from 19.4 ± 3.8 at baseline to 7.2 ± 5.4 at endpoint (*t* = 7.7,  *df* = 16,  *P* < 0.000001). This significant reduction in HDRS-21 total score was replicated, when the sample was limited to 12 MDD patients (19.8 ± 4.0 to 7.6 ± 5.3; *t* = 7.3, *df* = 11, *P* < 0.0001).  Figure 1 shows the mean scores over time on the HDRS-21 score of the sample (*n* = 17), based on the intent-to-treat analysis. This figure illustrates that the HDRS-21 score was reduced nearly by half within the first 4 weeks, followed by a further gradual reduction.

The CGI-S score decreased from 4.5 ± 0.7 (i.e., moderate to marked illness) at baseline to 2.5 ± 1.0 (i.e., borderline to mild illness) at endpoint (by Wilcoxon signed-rank test, *z* = −3.4, *P* = 0.001). This significant reduction in the CGI-S score was replicated when the sample was limited to 12 MDD patients (by Wilcoxon signed-rank test, *z* = −3.0, *P* = 0.003). The CGI-I score at endpoint indicated that 9 patients very much improved, 3 much improved, 3 minimally improved, and 2 showed no change. 

### 3.3. Safety 

Two patients dropped out from the study before the 12-week visit; one patient discontinued pramipexole at an early stage due to increased appetite and the other discontinued it after catching a common cold (Table 2). In total, adverse events likely to be caused by pramipexole were observed in 10 patients: nausea (*n* = 3), drowsiness (*n* = 3), orthostatic hypotension (*n* = 2), dry mouth (*n* = 1), insomnia (*n* = 1), agitation (*n* = 1), and increased appetite (*n* = 1). No serious events were seen except for persistent nausea in one patient which required an antiemetic drug. 

### 3.4. Summary of the Studies Investigating Efficacy of Adjunctive Dopamine Agonist in Refractory MDD 

Besides the present study, there have been six studies investigating the efficacy of adjunctive dopamine agonist in treatment-resistant unipolar MDD (Table 3). As for three studies that included depressed patients with bipolar disorder in addition to those with MDD [10–12], only the results for MDD patients are presented in this table. These studies overall showed substantial efficacy of dopamine agonist augmentation therapy in refractory depression, with the strongest evidence obtained for pramipexole. While most of the studies targeted stage I treatment-resistant depression, two recent studies including ours have observed marked efficacy of adjunctive dopamine agonist for stage II (or III) refractory depression. 

## 4. Discussion

The main finding was that many of our treatment-resistant depressed patients responded to adjunctive pramipexole treatment. This finding is in line with those of previous studies [8–11, 13]. Moreover, the present study, using a larger sample, confirmed the finding of Inoue et al. [13], that this effect can also be observed in stage II refractory patients. In addition, the present study suggested that adjunctive pramipexole may be effective even for stage III treatment-resistant depression. 

The majority of responders demonstrated the 50% or more reduction of HDRS-21 total score within the first 4 weeks of pramipexole treatment. Consistent with the present result, previous studies have shown that pramipexole augmentation in MDD brings a relatively rapid improvement in depressive symptoms [11, 13]. In treatment-resistant bipolar depression, two randomized controlled trials demonstrated that the addition of pramipexole to existing mood stabilizers resulted in a significant improvement in depressive symptoms [6, 7]. In MDD, there is one randomized controlled trial that investigated the effect of pramipexole, although that study did not examine “treatment-resistant” patients [18]. Thus, the evidence of pramipexole in refractory MDD has been scarce to date. Furthermore, efficacy of a dopamine agonist in refractory MDD patients whose degree of treatment resistance is explicitly defined as stage II or more has been examined only in one open trial [13], although the other studies investigating adjunctive pramipexole therapy in treatment-resistant depression may have included stage II MDD patients [8–12]. On the other hand, only one of the three dysthymic patients benefited from the pramipexole augmentation (Table 2). This suggests that pramipexole may be less effective in dysthymia than in pure MDD, although the small sample size does not allow any conclusions to be drawn regarding efficacy of pramipexole in dysthymia.

With respect to the dosage of pramipexole, a systematic review of the studies on pramipexole in mood disorders reported that the mean daily dose of pramipexole in the total 156 patients was 1.6 mg [19], which is almost identical to that in the present study. The final dosage of pramipexole varied widely between patients who showed response, from 0.25 to 3 mg/day. This indicates that pramipexole can exert its optimal therapeutic effect at a relatively low dose while higher doses may be needed in other cases. Since all of the four patients in whom pramipexole was increased up to 3 mg/day did not report serious adverse events, it may be suggested that the dosage be increased to 3.0 mg/day if the patient did not respond to lower dosage. Indeed, there is some evidence suggesting a dose-response relationship of pramipexole [19]. 

The mechanisms underlying the antidepressant effect of pramipexole are not elucidated. A recent neuroimaging study, however, showed that clinical improvement with pramipexole augmentation in bipolar depression was associated with a reduction in regional metabolism in orbitofrontal cortex, ventrolateral prefrontal cortex, and anteromedial prefrontal cortex [20]. This finding provides support for a role of the central dopaminergic system in the pathophysiology of depression since cerebral metabolic activity in these regions has been found to be elevated in depression [21, 22]. 

As for safety, no severe dopaminergic adverse events, such as delusions, hallucinations, and sleep attacks, were observed, although three patients experienced mild to moderate nausea. It is suggested that pramipexole has a lower risk of psychosis when used in depression than in Parkinson's disease [19], which may be attributable to relatively younger subjects in depression. In addition, no other serious side effects of pramipexole, including compulsive behavior and manic episodes, were seen in any of our patients. Our results therefore suggested good tolerability of pramipexole augmentation therapy for MDD. It should be noted, however, that the present 12-week trial was unable to examine any withdrawal effect of pramipexole. 

There were several limitations to the current study. First and foremost, this is an open study that does not have placebo or active control groups, which may have led to some potential biases such as observer bias. Second, the small sample size prevented us from conducting post-hoc multivariate analyses to control for or stratify by demographic/clinical variables. Third, our sample was heterogeneous in terms of diagnosis and treatment-resistant stage, although this might rather be advantageous in terms of ecological validity. Finally, concomitant medication was not standardized. 

In summary, pramipexole may be effective and relatively well tolerated in depressed patients who have failed to respond to previous medications. Future randomized controlled trials for treatment-resistant major depression are needed to prove the efficacy and safety of pramipexole.

## Figures and Tables

**Table 1:** Demographic characteristics and clinical variables at baseline.

Variable	Value
Diagnosis: bipolar II disorder/major depressive disorder	
Bipolar II disorder, *n *	5
Major depressive disorder, *n *	12

Stage of treatment-resistant depression	
Stage II	4
Stage III	13
Age, years: mean (SD)	36.2 ± 9.2
Gender: female, *n* (%)	10 (58.8)
Experience of hospitalizations: Yes, *n* (%)	6 (35.3)
History of suicidal attempt: Yes, *n* (%)	4 (23.5)
Family history of psychiatric disorder within first-degree relatives: Yes, *n* (%)	6 (35.3)
Age at onset, years: mean (SD)	28.1 ± 7.6
Age at first contact to psychiatric service, years: mean (SD)	30.2 ± 8.1
HDRS-21 total score: mean (SD)	19.4 ± 3.8
CGI-S score: median (range)	5 (3–6)

HDRS-21: 21-item version of the Hamilton Depression Rating Scale.

**Table 2:** Characteristics and outcome of the sample.

Patient number and DSM-IV diagnosis (Sex, Age)	Stage of treatment-resistant depression	Prior/concomitant medication (dosage: mg/day)	Follow-up visit (weeks)	Pramipexole dosage at endpoint (mg/day)	HDRS-21 decrease at endpoint (%)	Adverse events (reason for dropping out)
(1) BPII (M, 52)	Stage III	Amoxapine (125) Lithium (800)	12	1	100	None
			
(2) MDD, dysthymic disorder (F, 46)	Stage III	Fluvoxamine (50) Mirtazapine (15) Valproic acid (400)	4	0.25	27.3	Increased appetite
						(Increased appetite)
			
(3) BPII (M, 49)	Stage III	Amoxapine (75) Milnacipran (100) Sulpiride (150)	12	3	87.5	Orthostatic hypotension
			
			
(4) MDD (F, 41)	Stage III	Clomipramine (30) Lithium (600)	12	1	60.0	Nausea
			
(5) MDD (F, 38)	Stage III	Clomipramine (75) Sertraline (100)	12	1	58.8	Nausea
(6) MDD (M, 39)	Stage III		12	0.25	92.0	None
(7) MDD, dysthymic disorder (F, 28)	Stage III	Milnacipran (50) Aripiprazole (3)	12	1	83.3	None
			
(8) BPII (F, 30)	Stage III	Milnacipran (75) Aripiprazole (3) Valproic acid (300) Olanzapine (2.5)	12	3	6.3	Orthostatic hypotension
			
			
				
(9) MDD (M, 48)	Stage III	Duloxetine (20) Mirtazapine (30) Lithium (200) Mianserin (30)	12	2	41.2	Drowsiness
			
			
			
(10) MDD (M, 26)	Stage II	Paroxetine (40)	8	3	55.6	None (common cold)
(11) MDD (M, 32)	Stage III	Paroxetine (40) Sulpiride (600)	12	2	70.0	Nausea
			
(12) MDD, dysthymic disorder (M, 39)	Stage III	Paroxetine (40) Milnacipran (25)	12	3	17.4	Drowsiness
			
(13) MDD (F, 34)	Stage III	Clomipramine (150) Lithium (400) Sulpiride (150)	12	2	90.0	None
			
			
(14) MDD (F, 23)	Stage III	Amitriptyline (75) Sertraline (100) Valproic acid (400)	12	1.125	71.4	Agitation
						Insomnia
						Dry mouth
(15) BPII (F, 27)	Stage II	Mirtazapine (30) Carbamazepine (100)	12	1.5	31.3	Drowsiness
			
(16) MDD (F, 24)	Stage I	Sertraline (50)	12	1	73.3	None
(17) BPII (F, 40)	Stage III	Fluvoxamine (75) Paroxetine (20) Lithium (200)	12	1.5	81.3	None
			
			

MDD: major depressive disorder; BPII: bipolar II disorder; HDRS-21: 21-item version of the Hamilton Depression Rating Scale.

**Table 3:** Studies on the efficacy of adjunctive dopamine agonist in treatment-resistant unipolar major depressive disorder.

Study	*N* of subjects	Stage for treatment-resistant depression	Design	Dopamine agonist		Follow-up visit (weeks)	Efficacy (% responder)
				Drug	Dosage at endpoint (mg/day)		
The present study	12	mostly stage II and III	Open-label	pramipexole	1.47 ± 0.93	12	71^b^
Inoue et al. [8]	6	stage I	Open-label	bromocriptine	40.0^a^	6	67^b^
Izumi et al. [9]	20	stage I	Open-label	pergolide	0.59 ± 0.38^a^	4	40^c^
Sporn et al. [10]	20	stage I	Retrospective chart review	pramipexole	NA	NA	40^c^
Lattanzi et al. [11]	16	stage I	Open-label	pramipexole	0.95 ± 0.32^a^	16	64^d^
Cassano et al. [12]	7	stage I	Open-label	ropinirole	1.29	16	29^d^
Inoue et al. [13]	10	stage II	Open-label	pramipexole	1.3 ± 0.6^a^	8	60^d^

NA: not applicable.

^a^Maximum dosage.

^b^Defined as a reduction of 50% or more (from baseline to endpoint) in the Hamilton Depression Rating Scale total score.

^c^Defined as moderate to marked improvement (from baseline to endpoint) in the Clinical Global Impression-Improvement scale.

^d^Defined as a reduction of 50% or more (from baseline to endpoint) in the Montgomery-Asberg Depression Rating Scale total score.
